# Presentation at computed tomography (CT) scan of the thorax and first year diagnostic and treatment utilization among patients diagnosed with lung cancer

**DOI:** 10.1371/journal.pone.0181319

**Published:** 2017-07-14

**Authors:** Adam C. Powell, Amin J. Mirhadi, Bryan A. Loy, Laura E. Happe, James W. Long, Erin M. Kren, Amit K. Gupta

**Affiliations:** 1 HealthHelp, Houston, TX, United States of America; 2 Humana Inc., Louisville, KY, United States of America; Taipei Medical University, TAIWAN

## Abstract

**Background:**

As Medicare expands the use of computed tomography (CT) for diagnosing lung cancer, there is increased opportunity to diagnose lung cancer in asymptomatic patients. This descriptive study characterizes the disease-specific diagnostic and treatment services that patients with a positive diagnosis following CT received, stratified by presentation at CT.

**Methods:**

Patients who were diagnosed with lung cancer following CT in 2013, had no history of lung cancer, survived at least 1 year, were aged 55–80 years, and had Medicare Advantage insurance were included. Patients were grouped based upon presentation at CT: morbidities unrelated to lung cancer, classic lung cancer symptoms, and cancer syndromes. Patients with none of these factors were categorized into a no diagnoses/symptoms group. The type and intensity of services used in the year following the CT was reported for each group.

**Results:**

1,261 patients were included. Early treatment services were most common in the group with morbidities unrelated to lung cancer (13.7%) and least common in the cancer syndromes group (6.6%). Advanced treatment services were used by 47.3% of the cancer syndromes group versus 23.5% of the no diagnoses/symptoms group.

**Conclusions:**

The intensity of disease-specific diagnostic and treatment services varied by presentation at CT. Patients with no symptoms or morbidities at the time of CT less frequently received advanced interventions. Learning about the utilization patterns of others with a similar presentation at CT may help patients with positive lung cancer diagnoses engage in shared decision making and in norming their experiences against those of other similarly-situated patients.

## Introduction

Learning that one is likely to die of a disease with a somewhat known but non-immediate time course can often have a profound effect on how one chooses to live. [1] Such a diagnosis profoundly recalibrates life, introducing contingencies, and impacting important choices. [2] In order to help patients diagnosed with lung cancer make decisions and better anticipate what may happen to them, this study presents the disease-specific interventions that patients diagnosed with lung cancer following computed tomography (CT) experience in the year after their CT. The findings in the study are stratified according to presentation at CT, so that patients can learn about the experiences of others similar to themselves.

CTs are conducted both for screening and for investigating issues, such as chest pain or a persistent cough. An increasing number of patients are receiving CTs due to a combination of scientific findings and public policy changes. [3–6] In the National Lung Screening Trial (NLST), patients who were randomized to screening for lung cancer using CTs rather than chest radiographs experienced a significant, 20.0% relative reduction in mortality from lung cancer. [3] Early detection is important, as early delivery of thoracic irradiation has been shown to be associated with significantly lower rates of brain metastases, better progression-free survival, and better overall survival than later delivery of thoracic irradiation. [7] In response to the NLST, the Centers for Medicare & Medicaid (CMS) issued a National Coverage Determination mandating Medicare coverage of low-dose CT scanning in asymptomatic individuals aged 55–77 years who have a 30 pack-year smoking history and currently smoke or have quit within the past 15 years. [6] The U.S. Preventive Services Task Force (USPSTF) also recommends CT-based lung cancer screening according to similar parameters. [8] Thus, the standard of care has been changed in a manner which increases the number of patients receiving CTs to assess for the presence of lung cancer.

Using data from before Medicare’s National Coverage Determination, our study describes the disease-specific diagnostic and treatment services used by patients with a positive diagnosis of lung cancer, stratified by presentation at CT. Physician and hospital service utilization are not included, as these services are likely to be familiar to the patient. As only 18% of patients with lung cancer live for five or more years after diagnosis, the analysis focuses only on the experiences of patients during their first 365 days of care, starting from the day of CT. [9] For each group examined, the study describes the frequency with which each type of service was delivered and the number of episodes of care that were delivered on average. This information can be used to help physicians and newly diagnosed patients better understand the practical implications of a positive diagnosis.

## Methods

The objective of this descriptive study was to examine the frequency and quantity of disease-specific diagnostic and treatment services patients diagnosed with lung cancer utilize in the first year following their CT of the thorax, based upon their presentation at the CT. As this study was conducted as a part of Humana’s normal quality improvement operations, it did not meet the Department of Health and Human Service’s regulatory definition of research under 45 Code of Federal Regulations 46.102(d), and thus did not require Institutional Review Board approval. The authors have access to patient identifying information through the course of their daily job responsibilities and have accessed such data to complete this work.

### Sample selection

Medicare medical claims were extracted from the database of Humana, Inc., a healthcare company which in 2013 provided medical benefit plans to 12.0 million individuals across the nation. [10] To be included in the initial population extracted from the claims database, patients were required to have been continuously enrolled with the health plan between January 1, 2012 and December 31, 2014, to have had a CT of the chest without contrast (Current Procedure Terminology code 71250 or 71270) in 2013, to have had a claim indicating a lung cancer diagnosis subsequent to the CT, and to not have had any claims indicative of lung cancer in the 365 days prior to the 2013 CT. Patients found to have had metastatic cancer or a non-cancer diagnosis, such as solitary pulmonary nodule, were excluded from the population, as they may have had a disease other than primary lung cancer. Subsequent exclusions were made for patients who did not have a standard Medicare Advantage plan or switched from a commercial plan to Medicare during the study time frame, patients who had no evidence of follow-up care (having at least one claim in the focal areas in 365 days following the initial CT), patients who were not between 55–80 years of age in the study period, and patients who expired during the 365 days following the CT. No exclusions were made for false positives, as it is not possible to distinguish patients who declined treatment from false positives using claims data.

### Grouping by presentation at the time of CT

**Fig 1:**
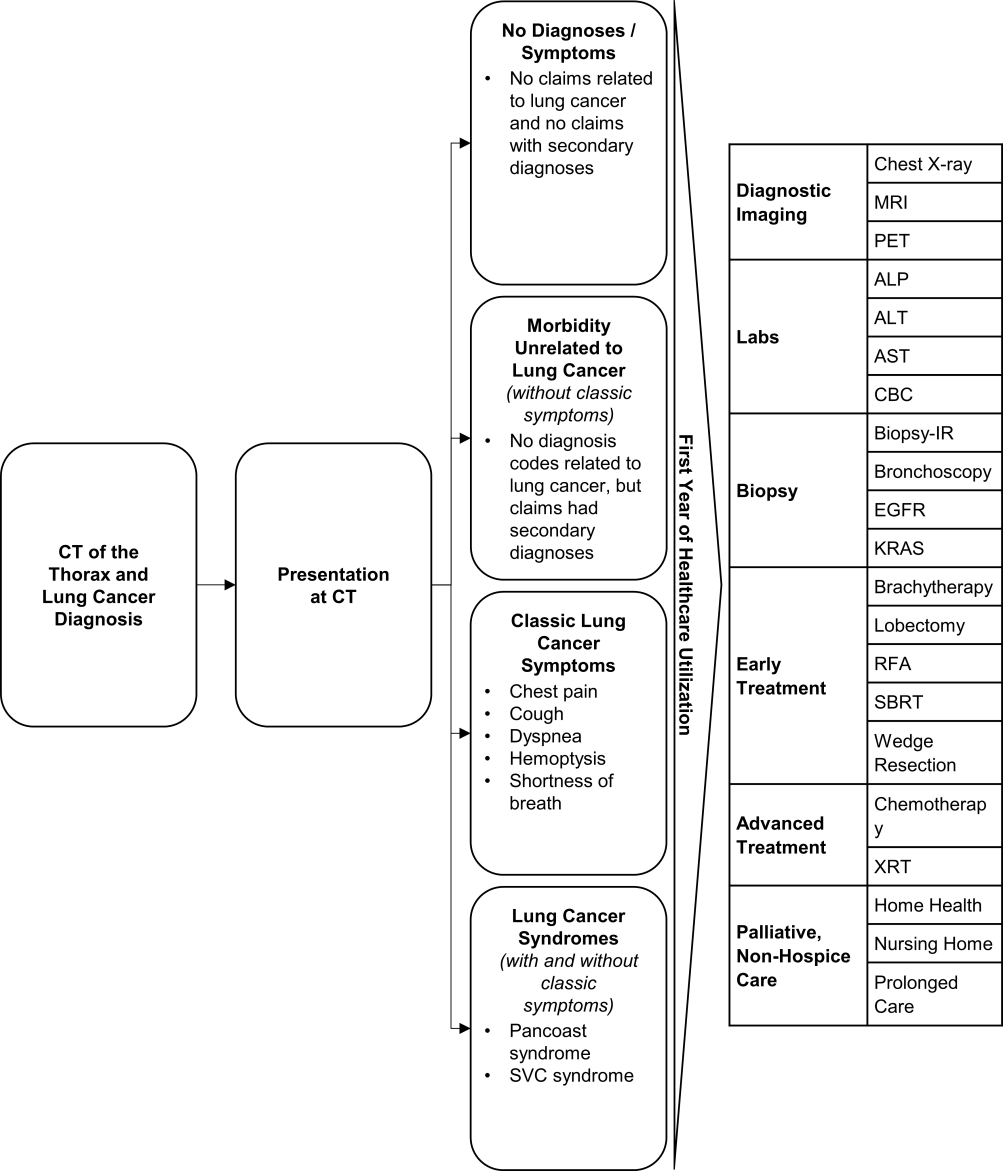
Study design diagram.

As shown in Fig 1, patients were sorted into four mutually-exclusive groups based upon a review of the primary and secondary diagnosis codes on the claims filed on the day of CT. The four groups were as follows: 1) no diagnoses or symptoms (no claims had diagnosis codes other than the primary diagnosis, which is required for billing), 2) morbidity unrelated to lung cancer (patients whose claims contained morbidities unrelated to lung cancer as secondary diagnoses), 3) classic lung cancer symptoms (patients whose claims included chest pain, cough, dyspnea, hemoptysis, or shortness of breath), and 4) lung cancer syndromes (patients whose claims included Pancoast syndrome or superior vena cava [SVC] syndrome). Group assignment occurred in a hierarchical fashion; patients with lung cancer syndromes were assigned to that group regardless of the presence of other morbidities. Next, those with classic lung cancer symptoms were assigned to that group. Afterwards, patients with morbidities unrelated to lung cancer were assigned to that group. Finally, the remaining patients were placed in the no diagnoses or symptoms group. Although Medicare was not covering lung cancer screenings using CT during the time of the study (2013), it is possible that some physicians may have chosen to order CTs for asymptomatic patients based upon the findings of the NLST, which was published in 2011. The CTs may also have been ordered to follow up on prior clinical findings unrelated to lung cancer.

### Intensity of disease-specific diagnostic and treatment services measurements

The claims incurred by patients in the 365 days following the CT were analyzed by group to determine the percentage of the individuals within each group who received each disease-specific diagnostic and treatment service at least once. In order to understand the intensity of care delivered, further analysis was performed to determine the average number of episodes of care being delivered to the recipients of a particular intervention. An episode of care was defined as a claim for services provided to one patient, using one modality, impacting one body part, on one day. No attempt was made to distinguish between claims that were related to lung cancer versus claims related to other illnesses. Day 1 of the 365-day survival period was defined as the date of the CT of the thorax. Since the timing of service delivery within Day 1 was not available, some of the services included in the analysis may have been provided a few hours before the initial CT. As clinical data were not available, it is not possible to observe the precise date of diagnosis. However, after a diagnosis is made, it may be reported on subsequent claims.

Twenty-one distinct healthcare services were included in the analyses (Fig 1). They represent services frequently used in the evaluation and treatment of lung cancer which are unlikely to be familiar to patients. Professional and hospital services associated with the care process (e.g. office visits, hospitalizations, and emergency room visits) were not included in the analysis. After diagnosis, a series of assessments are typically conducted to confirm and stage the cancer. Diagnostic imaging studies, such as chest x-rays (radiographs), magnetic resonance imaging (MRI), positron emission tomography (PET), are a common part of this process. These tests are often followed by labs, such as alkaline phosphatase (ALP), alanine aminotransferase (ALT), aspartate aminotransferase (AST), and complete blood count (CBC). Afterwards, biopsy and pathology studies are often conducted; these include bronchoscopy, biopsies conducted with radiological guidance, tests for epidermal growth factor receptor (EGFR), and tests for V-Ki-ras2 Kirsten rat sarcoma viral oncogene homolog (KRAS) mutations. KRAS was included, as it provides information on the probability of treatment response and because there are therapies under development which utilize the information it provides. [11]

The findings from these studies are used to inform treatments, which this study categorized into groups representing early treatment, advanced treatment, and palliative, non-hospice care. Early treatments include lobectomy, stereotactic body radiation therapy (SBRT), radiofrequency ablation (RFA), and wedge resection (both with and without brachytherapy). Advanced treatments include chemotherapy and radiation therapy (XRT). Palliative, non-hospice care includes home health, nursing home care, and prolonged care. Hospice care was not analyzed because it is not a component of Medicare Advantage plans. Coverage is not provided, as Original Medicare provides hospice coverage to people with Medicare Advantage plans. [12] As these services tend not to be mutually exclusive, a given patient could have been counted in more than one treatment category and have had more than one episode of care within a category. For instance, a patient might receive both an early treatment and an advanced treatment, or both early treatment and palliative care.

### Analyses

Demographic statistics on the age, gender, and regional location of the patients in each of the four groups were calculated. Chi-squared tests were conducted to determine if there was a significant association between group assignment and whether patients were under age 65 versus age 65 or older, female versus male, and located in the Midwest, South, or other regions. One-way analysis of variance (ANOVA) was conducted to determine if there was a significant association between group assignment and patient age. Results were then displayed as two tables: one table displaying the frequency with which members of each group experienced any episodes of care in each category, and another table displaying the average number of episodes received among group members who received at least one episode. Findings on the average number of episodes delivered were omitted when fewer than ten patients received a service in order to protect privacy and to avoid reporting numbers that may not be generalizable.

Results were calculated both for individual services and for categories of related services in the lung cancer diagnosis and treatment process. For instance, the “diagnostic imaging” category examined the frequency with which patients received a chest x-ray (radiograph), magnetic resonance image (MRI), or positron emission tomography (PET) scan. (CT is not included in the diagnostic imaging category, as all patients received a CT to be included in the study.) In calculating the frequency for the category, a patient who had received one or more of the aforementioned studies would be counted as having received diagnostic imaging. The same approach was followed in calculating the number of episodes of care delivered for each category.

To determine the percentage of patients who received any treatment, the early and advanced treatment categories were collapsed and reported. Individuals who receive a positive lung cancer diagnosis, diagnostic imaging, labs, and biopsies, but no treatment, may have received a false positive diagnosis. Some of the patients who did not receive treatment may have chosen to abstain from treatment or may have been in a physical condition that made them poor candidates for treatment.

## Results

### Study sample

The inclusion criteria yielded an eligible population of 15,861 patients. Exclusions were made for patients who were found to have had metastatic cancer or a non-cancer diagnosis (n = 12,682), patients who did not have a standard Medicare Advantage plan or switched from a commercial plan to Medicare during the study time frame (n = 110), patients with no evidence of follow-up care (n = 828), patients not between 55–80 years of age in the study period (n = 816), and patients who expired during the 365 days following the CT (n = 164). After all the exclusions were made, 1,261 patients were included in the sample. Within the population of 1,261 patients, 91 patients had lung cancer syndromes, 413 patients had classic lung cancer symptoms but no evidence of lung cancer syndromes, 621 patients had morbidities unrelated to lung cancer, and 136 had no lung cancer symptoms and no secondary diagnoses or symptoms on claims that occurred on the day of their lung cancer diagnosis. Demographic information for each of the four groups is presented in Table 1. The groups did not have significantly different mean ages (P = .26), percentages of patients under the age of 65 (P = .40), or percentages of female patients (P = .45). There was not a significant association between group assignment and geographic origin (P = .59). All of the groups contained a disproportionate number of patients from the Midwest and the South; a bias attributable to the market footprints of the health plans to which the patients belonged.

**Table 1:** Demographics.

	No Diagnoses / Symptoms	Morbidity Unrelated to Lung Cancer	Classic Lung Cancer Symptoms	Lung Cancer Syndromes	Overall
**n**	136	621	413	91	1,261
**Average Age**	71.4	72.0	71.6	72.8	71.9
**% Under 65**	11.8%	9.7%	12.1%	7.7%	10.5%
**% Female**	44.1%	50.7%	52.1%	50.5%	50.4%
**% Midwest Region**	32.4%	41.3%	41.2%	37.4%	40.0%
**% South Region**	58.1%	49.8%	50.1%	54.9%	51.2%
**% Other Region**	9.6%	9.0%	8.7%	7.7%	8.8%

**Fig 2:**
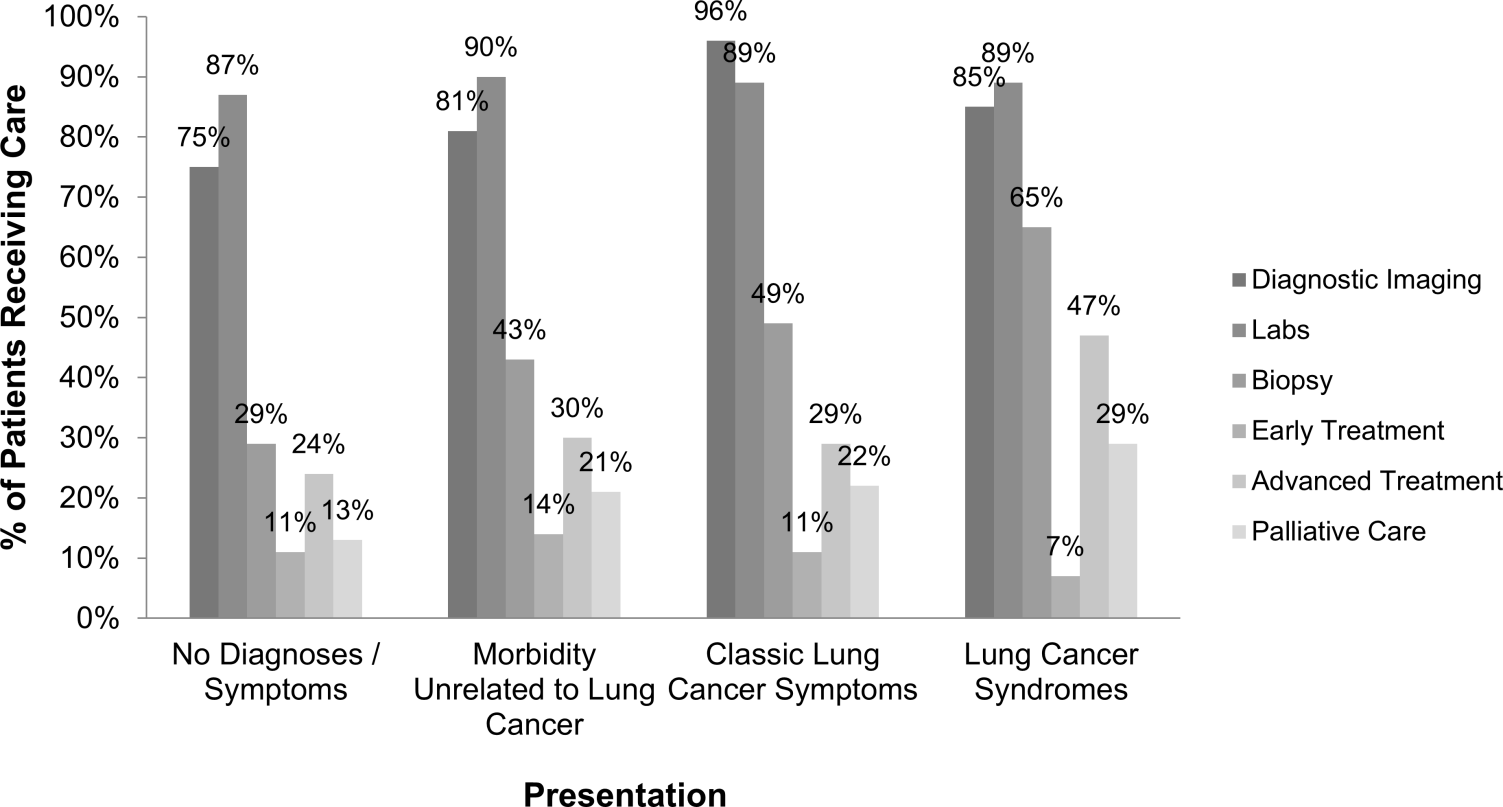
Healthcare received during first year post-diagnosis, by presentation at the time of CT.

The frequency with which each category of healthcare service was used by each group is displayed in Fig 2. Patients who presented with lung cancer syndromes at the time of the CT were more likely to receive additional diagnostic imaging, labs, biopsy, advanced treatment, and palliative care than patients who presented with no diagnoses or symptoms. The use of early treatment was nearly twice as common among patients with no diagnoses or symptoms at the time of the CT as it was in patients with lung cancer symptoms at diagnosis.

### Disease-specific healthcare services: Work-up

Work-up utilization was reported separately for diagnostic imaging, labs, and biopsy. As shown in Table 2, the rates at which specific services were utilized varied dramatically between groups. Diagnostic imaging other than CT (everyone had a CT) was utilized by 85.6% of the population, ranging from 75.0% of those with no diagnoses or symptoms to 95.6% of those with classic lung cancer symptoms. The most common additional imaging study conducted was a chest x-ray (82.5% of the population). As indicated in Table 3, the number of episodes of each diagnostic imaging test used did not vary dramatically between the groups. The average number of diagnostic images performed per patient ranged from 4.7 in the no diagnoses or symptoms group to 6.0 in the lung cancer syndromes group. The variation was largely driven by the number of chest x-rays performed.

**Table 2:** Frequency of disease-specific healthcare service utilization.

		No Diagnoses / Symptoms (n = 136)	Morbidity Unrelated to Lung Cancer (n = 621)	Classic Lung Cancer Symptoms (n = 413)	Lung Cancer Syndromes (n = 91)	Total (n = 1,261)
**Diagnostic Imaging**	**Chest X-ray**	69.1%	77.3%	94.7%	82.4%	**82.5%**
	**MRI**	0.7%	0.2%	0.0%	0.0%	**0.2%**
	**PET**	39.0%	44.6%	44.3%	47.3%	**44.1%**
	**Total**	**75.0%**	**81.5%**	**95.6%**	**84.6%**	**85.6%**
**Labs**	**ALP**	0.7%	4.8%	4.4%	4.4%	**4.2%**
	**ALT**	5.9%	9.8%	9.2%	9.9%	**9.2%**
	**AST**	6.6%	8.9%	7.5%	9.9%	**8.2%**
	**CBC**	86.0%	88.9%	89.1%	89.0%	**88.7%**
	**Total**	**86.8%**	**89.9%**	**89.3%**	**89.0%**	**89.3%**
**Biopsy**	**Biopsy-IR**	19.9%	30.3%	30.5%	35.2%	**29.6%**
	**Bronchoscopy**	14.0%	20.1%	27.1%	42.9%	**23.4%**
	**EGFR**	5.1%	5.8%	4.6%	7.7%	**5.5%**
	**KRAS**	0.7%	1.0%	1.0%	0.0%	**0.9%**
	**Total**	**29.4%**	**42.7%**	**48.7%**	**64.8%**	**44.8%**
**Early Treatment**	**Brachytherapy**	0.7%	0.2%	0.0%	0.0%	**0.2%**
	**Lobectomy**	6.6%	9.0%	7.7%	5.5%	**8.1%**
	**RFA**	0.0%	0.2%	0.0%	0.0%	**0.1%**
	**SBRT**	2.2%	2.9%	1.7%	1.1%	**2.3%**
	**Wedge Resection**	2.2%	2.7%	2.2%	0.0%	**2.3%**
	**Total**	**11.0%**	**13.7%**	**10.9%**	**6.6%**	**12.0%**
**Advanced Treatment**	**Chemotherapy**	16.2%	21.7%	23.2%	37.4%	**22.8%**
	**XRT**	14.7%	17.4%	19.6%	36.3%	**19.2%**
	**Total**	**23.5%**	**30.0%**	**28.8%**	**47.3%**	**30.1%**
**Palliative, Non-Hospice Care**	**Home Health**	11.0%	16.4%	18.2%	20.9%	**16.7%**
	**Nursing Home**	2.9%	9.3%	8.2%	11.0%	**8.4%**
	**Prolonged Care**	1.5%	0.6%	1.7%	2.2%	**1.2%**
	**Total**	**13.2%**	**20.8%**	**21.8%**	**28.6%**	**20.9%**

**Table 3:** Mean number of episodes of healthcare service utilization per patient among patients receiving 1+ episodes.

		No Diagnoses / Symptoms (n = 136)		Morbidity Unrelated to Lung Cancer (n = 621)		Classic Lung Cancer Symptoms (n = 413)		Lung Cancer Syndromes (n = 91)		Total (n = 1,261)	
	Unit	Eps.	n	Eps.	n	Eps.	n	Eps.	n	Eps.	n
**Diagnostic Imaging**	**Chest X-ray**	4.3	**94**	5.1	**480**	5.0	**391**	5.3	**75**	**5.0**	**1,040**
	**MRI**	*	*	*	*	*	*	*	*	*	*
	**PET**	1.3	**53**	1.3	**277**	1.3	**183**	1.4	**43**	**1.3**	**556**
	**Total**	**4.7**	**102**	**5.5**	**506**	**5.5**	**395**	**6.0**	**77**	**5.5**	**1,080**
**Labs**	**ALP**	*	*	2.6	**30**	1.6	**18**	*	*	**2.3**	**53**
	**ALT**	*	*	1.8	**61**	1.5	**38**	*	*	**1.7**	**116**
	**AST**	*	*	1.9	**55**	1.5	**31**	*	*	**1.9**	**104**
	**CBC**	4.9	**117**	5.9	**552**	6.2	**368**	9.3	**81**	**6.1**	**1,118**
	**Total**	**5.1**	**118**	**6.3**	**558**	**6.5**	**369**	**9.9**	**81**	**6.5**	**1,126**
**Biopsy**	**Biopsy-IR**	1.1	**27**	1.1	**188**	1.2	**126**	1.1	**32**	**1.2**	**373**
	**Bronchoscopy**	1.4	**19**	1.2	**125**	1.3	**112**	1.2	**39**	**1.2**	**295**
	**EGFR**	*	*	1.0	**36**	1.0	**19**	*	*	**1.0**	**69**
	**KRAS**	*	*	*	*	*	*	*	*	**1.0**	**11**
	**Total**	**1.6**	**40**	**1.6**	**265**	**1.6**	**201**	**1.5**	**59**	**1.6**	**565**
**Early Treatment**	**Brachytherapy**	*	*	*	*	*	*	*	*	*	**2**
	**Lobectomy**	*	*	1.0	**56**	1.0	**32**	*	*	**1.0**	**102**
	**RFA**	*	*	*	*	*	*	*	*	*	**1**
	**SBRT**	*	*	3.9	**18**	*	*	*	*	**3.8**	**29**
	**Wedge Resection**	*	*	1.0	**17**	*	*	*	*	**1.0**	**29**
	**Total**	**1.6**	**15**	**1.8**	**85**	**1.5**	**45**	*	**6**	**1.6**	**151**
**Advanced Treatment**	**Chemotherapy**	7.7	**22**	8.1	**135**	9.5	**96**	10.7	**34**	**8.9**	**287**
	**XRT**	22.3	**20**	20.5	**108**	25.7	**81**	28.1	**33**	**23.4**	**242**
	**Total**	**19.2**	**32**	**17.8**	**186**	**25.2**	**119**	**30.0**	**43**	**21.6**	**380**
**Palliative, Non-Hospice Care**	**Home Health**	8.7	**15**	11.0	**102**	11.5	**75**	13.0	**19**	**11.2**	**211**
	**Nursing Home**	*	*	7.8	**58**	5.0	**34**	5.7	**10**	**6.5**	**106**
	**Prolonged Care**	*	*	*	*	*	*	*	*	**1.0**	**15**
	Total	8.1	18	12.2	129	11.5	90	11.8	26	11.7	263

(* = redacted to protect privacy, as there were fewer than 10 cases)

Labs were performed on 89.3% of those diagnosed with lung cancer, with the rate of lab use differing 3.1% between the groups with the lowest and highest rates. Lab usage did not vary substantially with presentation at CT, except in the case of CBC. CBC use ranged between an average of 4.9 tests among those with no diagnoses or symptoms to 9.3 tests among those with lung cancer syndromes.

Biopsy rates varied substantially between the groups. The lung cancer syndromes group had a 64.8% biopsy rate, while patients without diagnoses or symptoms had only a 29.4% biopsy rate. Patients with lung cancer syndromes were most likely to receive each of the tests, except for KRAS (for which all groups had a rate below 1.0%). For each of the tests, no group on average received more than 1.4 episodes of testing.

### Disease-specific healthcare services: Treatment

Of the 1,261 patients diagnosed with lung cancer, 36.6% (461/1,261) went on to receive early or advanced treatment (collapsed, data not shown). Treatment was received by 29.4% (40/136) of those with no diagnoses or symptoms, 37.4% (232/621) of those with morbidities unrelated to lung cancer, 34.6% (143/413) of those with classic lung cancer symptoms, and 50.5% (46/91) of those with lung cancer syndromes.

The use of early treatment varied dramatically by presentation at CT. Only 6.6% of patients with lung cancer syndromes at diagnosis received early treatment, while 13.7% of patients with morbidities unrelated to lung cancer received early treatment. In all groups, the most common early treatment was lobectomy. As a result of the low frequency of use of many of the early treatments, it has been often necessary to omit the average number of episodes of care delivered to protect privacy.

In contrast, patients with lung cancer syndromes at CT were the most likely to receive advanced treatment, as 47.3% received it. The other groups received advanced treatment at rates at or below 30.0%. Although the frequencies were similar, chemotherapy was slightly more commonly used than radiation therapy in each of the groups.

Utilization rates for palliative, non-hospice care were lowest for those healthiest at the time of CT, ranging from 13.2% for the no diagnoses or symptoms group to 28.6% for the lung cancer syndromes group. Patients who presented with no diagnoses or symptoms used the fewest episodes of home health, an average of 8.7, while patients who presented with lung cancer symptoms used the most episodes, an average of 13.0.

## Discussion

As the use of CT for screening and evaluating potential lung cancer becomes more prevalent in the wake of the CMS coverage decision, a growing number of patients may be diagnosed with lung cancer and wonder what the future holds for them. The information provided in Tables 2 and 3 of this study offers perspective for patients from the vantage point of the time of CT. The existing literature on lung cancer diagnosis by CT provides insights into issues such as the likelihood of a false positive [3, 13–15], the rate of malignant disease by size of non-calcified nodule [16], and life expectancy [5]. This paper extends the literature by providing insights into the potentially unfamiliar diagnostics and treatments a patient is likely to experience after being diagnosed with lung cancer.

The findings of this study may additionally help health insurers project some forms of resource utilization. Caution should be taken in interpreting the findings, as the study was conducted using observational data from before the CMS and USPSTF guidelines were released. The release of these guidelines and the promotion of CT screening for lung cancer may change the characteristics of the cohort being screened.

Patients recently diagnosed with lung cancer may have anxiety regarding their out-of-pocket exposure to the cost of the various healthcare services used during the lung cancer diagnosis and treatment process. Patients with Medicare Advantage are limited in their annual exposure to the costs. Beginning in 2011, a mandatory $6,700 maximum out-of-pocket limit on total enrollee cost sharing for Part A and B services was implemented for Medicare Advantage plans. [17] Out-of-pocket spending includes payments towards the deductible, coinsurance, and copayments, but not health plan premiums. While some patients may not need to pay costs reaching their out-of-pocket maximum, whether this is the case is codetermined by the nature of the cancer, the design of the patient’s health plan, and the extent to which care is provided by clinicians and sites of care with in-network contracts with the health plan.

### Limitations

This study was conducted using claims, and there are a number of limitations that are inherent to this approach. Physicians vary in their diligence in documenting the condition of patients when they write orders. Even factors such as the number of other patients being contemporaneously treated may impact the completeness of claims. [18] Thus, some of the patients examined by this study may have had more morbidities and cancer symptoms at the time of diagnosis than was reported in the claims data (misclassification bias), and likewise may have utilized more care than is reflected in the claims data (underreporting bias). Although all claims were included in the study, there may have been under-reporting of services used in an inpatient setting, as there is evidence that the use of Diagnosis-Related Groups (DRGs) for inpatient billing may lead to the under-reporting of CTs, MRIs, and other services, as reporting such services may not lead to increased revenue under DRG payments. [19] Hospital and physician services were outside the scope of the analysis. Furthermore, as this study reported all utilization for disease-specific diagnostic and treatment services, some of the claims included may have been unrelated to lung cancer.

Timing issues add some imprecision to the findings. As claims do not report the findings of diagnostic imaging, labs, and biopsies, it is only possible to infer clinical findings by observing the diagnoses listed on subsequent claims. Thus, it is not possible to precisely determine the date that a patient received a diagnosis. Moreover, the precise timing of claims on the day of the CT is not known. While the one year window examined by this study starts with a CT of the thorax, it is possible that other diagnostics were conducted before the CT, on the day that the CT was performed. These same day diagnostics are included in the analysis.

The use of claims data from health plans offered by only one company may impact the generalizability of the findings. While the company has a national presence, its membership is concentrated in the Midwest and South, with a limited presence in the Northeast. Thus, any regional practice variation that exists may bias the results. Furthermore, people with Medicare Advantage plans may utilize a different approach to healthcare decision making than people with Original Medicare plans, as picking a Medicare Advantage plan requires active decision making.

Another limitation of the study is that only the first 365 days of claims post-CT among patients who lived with a positive diagnosis for that period were examined. Patients who died within a year of the CT have been removed from the sample. This population has been removed because it contained patients who received treatment for differing lengths of time. As the claims data did not contain information on the stage of cancer patients were assigned at diagnosis, it is unknown whether this exclusion has biased the study towards the lower intensity of services that would be expected with earlier stage cancers. Furthermore, as false positives were not excluded from the populations, the findings may under-represent what is experienced by patients with true positive diagnoses of cancer. The rates of false positives may have varied between the four groups, perhaps explaining why the patients in the “no diagnoses / symptoms” group were the least likely to receive diagnostic imaging, labs, and biopsies. Nonetheless, because patients are likely unsure of whether they have a true or false positive case of lung cancer when they are diagnosed, the findings of the study may be useful to individuals newly diagnosed with cancer who present clinically as likely to live for at least a year post-diagnosis.

Finally, there are ongoing efforts to advance the treatment of lung cancer. While the results of this study are representative of the first-year experiences of a population that received CTs in 2013, future patients may have different experiences. In 2015 and 2016, the Food and Drug Administration (FDA) approved multiple new drugs for the treatment of metastatic non-small cell lung cancer. [20] As a result, future patients may use differing combinations of services as the options available to them evolve.

## Conclusions

The disease-specific diagnostic and treatment service utilization patterns that patients with lung cancer experience differ according to presentation at CT. Learning about the utilization patterns of others diagnosed with a similar presentation may help those with positive lung cancer diagnoses conceptualize the implications of their diagnosis. Doing so may be helpful when engaging in shared decision making and when norming experiences against those of other similarly-situated patients.

As a larger number of patients receive CTs of the thorax due to the decisions of CMS and the USPSTF, there may be an increasing number of opportunities to diagnose lung cancer in asymptomatic patients. Patients without any diagnoses or symptoms at the time of CT were found to be less likely to receive advanced interventions or palliative, non-hospice care in the year following the CT than were patients with related and unrelated morbidities. Thus, increased performance of CT of the thorax on asymptomatic patients may identify more patients in need of less intensive interventions.
